# Coordinated Primary-Care Access in Rural and Suburban Alberta, with a Contextual Comparison to Rural Wyoming: A Systematic Review and Narrative Synthesis of Community Pharmacist–Family Physician Care Models

**DOI:** 10.3390/pharmacy14040098

**Published:** 2026-07-02

**Authors:** Tomasz Karczewski, Jennifer M. L. Stephens, Dawid Karczewski, Sahar Feizizadeh, Dhwani Dixit, Mihaela Olsen

**Affiliations:** 1 Cranston Ridge Medical Clinic, Calgary, AB T3M 3A9, Canada; tomasz@cranstonridgemedical.com (T.K.); mihaela_emily@yahoo.com (M.O.); 2 Fay W. Whitney School of Nursing, University of Wyoming, Laramie, WY 82071, USA; jsteph35@uwyo.edu; 3 Cranston Smart Drug Mart, Calgary, AB T3M 3A9, Canada; sahar@cranstonridgemedical.com (S.F.); dhwanidixit17@gmail.com (D.D.)

**Keywords:** primary care access, rural health, suburban health, Alberta, Wyoming, community pharmacists, family physicians, physician-pharmacist collaboration, pharmacist prescribing, medication management, chronic disease, emergency department utilization, narrative synthesis

## Abstract

**Background/Objectives:** Primary-care access in Alberta, Canada, is shaped by geography, attachment, timeliness, continuity, and local service capacity. Rural communities may face travel burden, workforce fragility, and intermittent services, whereas suburban communities may have nearby facilities but still experience delayed access, low attachment, and fragmented episodic care. Rural Wyoming has some similar geographic and workforce constraints, although the jurisdictions differ in financing, regulation, and pharmacist scope. This systematic review and narrative synthesis examined evidence on coordinated community pharmacist–family physician care in rural and suburban Alberta and considered, separately, the contextual relevance of the findings to rural Wyoming and comparable frontier settings. **Methods:** We searched PubMed/MEDLINE, Embase, Scopus, CINAHL, and the Cochrane Library using controlled vocabulary and free-text terms to identify English-language peer-reviewed studies and practice-relevant evidence published from 1 January 2010 to 19 April 2026. Two authors screened titles/abstracts and full texts and resolved decisions by consensus. Methodological appraisal used design-appropriate Critical Appraisal Skills Programme criteria, and outcome-level certainty was considered using GRADE domains. **Results:** Thirty-four eligible peer-reviewed or practice-evaluation records were included in the narrative synthesis, and seven official contextual or methodological sources supported jurisdictional interpretation. Evidence was strongest for hypertension, cardiovascular risk reduction, medication management, and chronic disease monitoring. No included study directly compared the same intervention in Alberta and Wyoming; evidence for emergency-department effects and equivalent effectiveness across settings was limited. **Conclusions:** Coordinated pharmacist–family physician care may extend access to selected medication-related and chronic disease services when supported by documentation, referral, follow-up, and red-flag protocols. Application to Wyoming should be treated as a context-dependent proposition requiring local adaptation and prospective evaluation, not as demonstrated transferability or a substitute for physician-led longitudinal primary care.

## 1. Introduction

Access to primary care is a foundational determinant of health-system performance as early first-contact care, longitudinal relationships, preventive counselling, medication management, and chronic disease monitoring influence downstream emergency-department use, hospitalization, patient experience, and equity [1,2,3]. Access to primary care is not simply the physical presence of a clinician. The patient-centred access framework proposed by Levesque and colleagues conceptualizes access at the interface between health-system dimensions and population abilities, including approachability, acceptability, availability and accommodation, affordability, and appropriateness [1]. This multidimensional model is important when evaluating rural, suburban, and frontier communities as different communities can experience different access failures within the same health system.

Rural healthcare in Canada has long been associated with workforce shortages, long travel distances, service fragility, and reduced availability of specialized and allied health services [3,4]. Alberta-specific evidence illustrates how geography can become a practical access barrier. Spatial-access studies of people with osteoarthritis in Alberta identified rural–urban differences in local and non-local primary-care use and showed that rural and remote communities may experience substantially longer realized travel times to general practitioners, orthopedic surgeons, and physiotherapists than metropolitan communities [5,6]. These studies are disease-specific, but they demonstrate a broader health-planning principle: local access, travel time, and continuity should be measured directly rather than assumed from the presence of a regional service.

Suburban Alberta presents a different access problem. Suburban communities usually have greater physical proximity to clinics, pharmacies, urgent-care centres, and metropolitan health infrastructure than remote communities, but proximity does not guarantee attachment, same-day access, continuity, or appropriateness. A large Canadian survey found persistent gaps in primary-care attachment and urgent appointment access, and Alberta policy analyses have described a substantial post-pandemic shortage of family physician services [7,8]. Therefore, suburban access problems may be expressed less as distance and more as delayed appointments, low attachment, walk-in dependence, repeated episodic care, and poor information flow between providers.

Wyoming was selected as a contextual U.S. frontier comparator, not as a jurisdiction assumed to be equivalent to Alberta. Its dispersed communities, travel burden, workforce pressures, and reliance on local professional relationships provide a useful setting in which to consider whether mechanisms identified in Alberta may be relevant. Important differences in population, insurance, reimbursement, regulation, and pharmacist scope, however, prevent any assumption of comparable effects. Health Resources and Services Administration data listed 47 primary-care Health-Professional Shortage Area designations in Wyoming as of 31 March 2026, covering 243,465 people and estimating that 30 additional practitioners would be needed to remove the designations [9]. The Wyoming Office of Rural Health supports workforce and shortage-designation analysis, technical assistance, collaboration, and access initiatives for underserved populations [10]. Wyoming-specific qualitative and policy research also identifies trust, communication, time with clinicians, weather, distance, cost, and payer constraints as relevant to rural access [11,12]. These sources establish context for cautious interpretation; they do not provide comparative intervention evidence.

Community pharmacists are positioned at an important interface between these access problems. They are widely distributed, frequently available without appointment, and often contacted more frequently than physicians by patients requiring medications, monitoring, or self-care advice. United States Medicare data show that older adults visit community pharmacies more often than they encounter primary-care physicians, with the difference particularly large in rural nonmetropolitan areas [13]. United States geographic-access studies also show that most people live near a community pharmacy, although rural access remains more vulnerable than suburban or urban access and depends heavily on independent or regional pharmacies [14,15]. In Alberta, pharmacist scope includes prescribing authorities, medication adaptation and renewal, injection services, laboratory test ordering and interpretation, and other clinical services for appropriately authorized pharmacists [16,17,18]. Recent Canadian commentary has also described the emergence of pharmacist primary-care clinics [19].

The question is not whether pharmacists should replace family physicians. A more clinically relevant question is whether coordinated pharmacist–family physician care can extend primary-care capacity while preserving continuity, medication safety, and diagnostic escalation. Coordination is important because faster access may increase fragmentation when a pharmacy encounter is disconnected from the patient’s regular clinic, medication list, or follow-up plan [5,20]. This review therefore addressed two related but distinct questions: What evidence supports coordinated pharmacist–family physician care for access, medication management, chronic disease monitoring, continuity, emergency-department utilization, medication safety, and equity? What contextual similarities and differences should be considered before any adaptation to rural Wyoming? The second question was treated as interpretive and hypothesis-generating, not as a test of comparative effectiveness.

## 2. Materials and Methods

### 2.1. Protocol and Review Question

A structured review protocol was developed before synthesis and the review was registered with PROSPERO (registration number CRD420261414639; registered 3 June 2026). The full internal protocol was developed for the review team and was not separately published. The review was conducted as a systematic review with narrative synthesis because the evidence included heterogeneous study designs, jurisdictions, access indicators, intervention models, and outcomes. The manuscript was prepared with reference to the PRISMA 2020 statement where applicable to a narrative synthesis without meta-analysis [21]. The completed PRISMA 2020 checklist is provided in Supplementary File S1. Evidence included in the narrative synthesis was kept separate from official policy, regulatory, shortage-area, and methodological sources used only for contextual interpretation. The review was conducted as a systematic review with narrative synthesis because the evidence included heterogeneous study designs, jurisdictions, access indicators, intervention models, and outcomes.

The primary review question was among adults and families living in rural and suburban Alberta and in relevant Canadian or U.S. rural/frontier settings, how does coordinated community pharmacist–family physician care, compared with usual physician-centred or fragmented episodic pathways, influence timely access, medication management, chronic disease monitoring, continuity, emergency-department utilization, medication safety, and equity? A secondary question examined which contextual similarities and differences should be considered before adapting evidence-informed components to rural Wyoming. This secondary question was not treated as a direct Alberta–Wyoming comparative effectiveness analysis.

### 2.2. Eligibility Criteria

Eligibility criteria were designed to capture Alberta-specific access evidence, Canadian pharmacy-practice evidence, and relevant U.S. rural/frontier evidence. The prespecified contemporary publication window for peer-reviewed and practice-relevant evidence was 1 January 2010 to 19 April 2026. Earlier landmark primary-care, physician–pharmacist collaboration, and medication-safety studies were retained only when needed for conceptual framing. Official policy, regulatory, shortage-area, and reporting sources were classified separately and were not treated as intervention-effect evidence. The eligibility criteria and source categories are summarized in Table 1.

### 2.3. Information Sources and Search Strategy

Searches were conducted in PubMed/MEDLINE, Embase, Scopus, CINAHL, and the Cochrane Library; all final database searches were completed on 19 April 2026. Searches combined controlled vocabulary and free-text terms for primary-care access, rural health, suburban or peri-urban settings, frontier settings, Alberta, Canada, Wyoming, community pharmacists, pharmacist prescribing, physician–pharmacist collaboration, pharmacy clinics, chronic disease management, medication review, medication safety, emergency-department use, and continuity of care. Reference lists of included reviews and key studies were checked for additional eligible publications. During revision, official Alberta and Wyoming contextual sources were rechecked on 23 June 2026; this did not add intervention-effect studies or change the synthesis set. Grey literature was restricted to official health-system, regulatory, shortage-area, or reporting sources and was classified separately from intervention-effect evidence.

The PubMed/MEDLINE core search string was (“Primary Health Care”[Mesh] OR “Health Services Accessibility”[Mesh] OR access*[tiab] OR attachment[tiab] OR continuity[tiab] OR “care coordination”[tiab] OR “emergency department”[tiab] OR “ambulatory care sensitive”[tiab]) AND (rural[tiab] OR remote[tiab] OR suburban[tiab] OR periurban[tiab] OR “peri-urban”[tiab] OR frontier[tiab] OR Alberta[tiab] OR Canada[tiab] OR Wyoming[tiab] OR “United States”[tiab]) AND (pharmacist*[tiab] OR pharmacy[tiab] OR “community pharmacy”[tiab] OR “pharmacist prescribing”[tiab] OR “physician pharmacist”[tiab] OR “family physician pharmacist”[tiab] OR “collaborative practice”[tiab] OR “minor ailment”[tiab] OR “common ailment”[tiab]) AND (“2010/01/01”[Date-Publication]: “2026/04/19”[Date-Publication]).

The Embase core string was (primary health care/exp OR health care access/exp OR access*:ti,ab OR attachment:ti,ab OR continuity:ti,ab OR ‘care coordination’:ti,ab OR ‘emergency department’:ti,ab OR ‘ambulatory care sensitive’:ti,ab) AND (rural:ti,ab OR remote:ti,ab OR suburban:ti,ab OR periurban:ti,ab OR ‘peri-urban’:ti,ab OR frontier:ti,ab OR Alberta:ti,ab OR Canada:ti,ab OR Wyoming:ti,ab OR ‘United States’:ti,ab) AND (pharmacist/exp OR pharmacy/exp OR pharmacist*:ti,ab OR ‘community pharmacy’:ti,ab OR ‘pharmacist prescribing’:ti,ab OR ‘physician pharmacist’:ti,ab OR ‘collaborative practice’:ti,ab OR ‘minor ailment’:ti,ab OR ‘common ailment’:ti,ab) AND [2010–2026]/py AND [humans]/lim.

The Scopus core string was TITLE-ABS-KEY (access* OR attachment OR continuity OR “care coordination” OR “emergency department” OR “ambulatory care sensitive”) AND TITLE-ABS-KEY (rural OR remote OR suburban OR periurban OR “peri-urban” OR frontier OR Alberta OR Canada OR Wyoming OR “United States”) AND TITLE-ABS-KEY (pharmacist* OR pharmacy OR “community pharmacy” OR “pharmacist prescribing” OR “physician pharmacist” OR “collaborative practice” OR “minor ailment” OR “common ailment”) AND PUBYEAR > 2009 AND PUBYEAR < 2027.

The CINAHL core string was (MH “Primary Health Care” OR MH “Health Services Accessibility” OR access* OR attachment OR continuity OR “care coordination” OR “emergency department”) AND (rural OR remote OR suburban OR periurban OR “peri-urban” OR frontier OR Alberta OR Canada OR Wyoming OR “United States”) AND (pharmacist* OR pharmacy OR “community pharmacy” OR “pharmacist prescribing” OR “physician pharmacist” OR “collaborative practice” OR “minor ailment” OR “common ailment”), limited to human studies published from 2010 to 2026.

The Cochrane Library core string was (rural OR remote OR suburban OR frontier OR Alberta OR Canada OR Wyoming OR “United States”) AND (primary care OR access OR continuity OR coordination OR emergency department) AND (pharmacist OR pharmacy OR “community pharmacy” OR prescribing OR “physician pharmacist” OR “collaborative practice” OR “minor ailment” OR “common ailment” OR “pharmacy care clinic”).

### 2.4. Study Selection and Data Extraction

Two authors (D.K. and T.K.) screened records at title/abstract and full-text stages against the prespecified eligibility criteria. Records were assessed for relevance to primary-care access, rural/suburban/frontier context, community pharmacy, pharmacist–family physician coordination, medication management, chronic disease care, emergency-department use, and Alberta–Wyoming contextual relevance. Decisions were discussed and finalized by consensus. A formal inter-reviewer agreement statistic was not calculated. No automation tools were used to make eligibility decisions.

**Figure 1:**
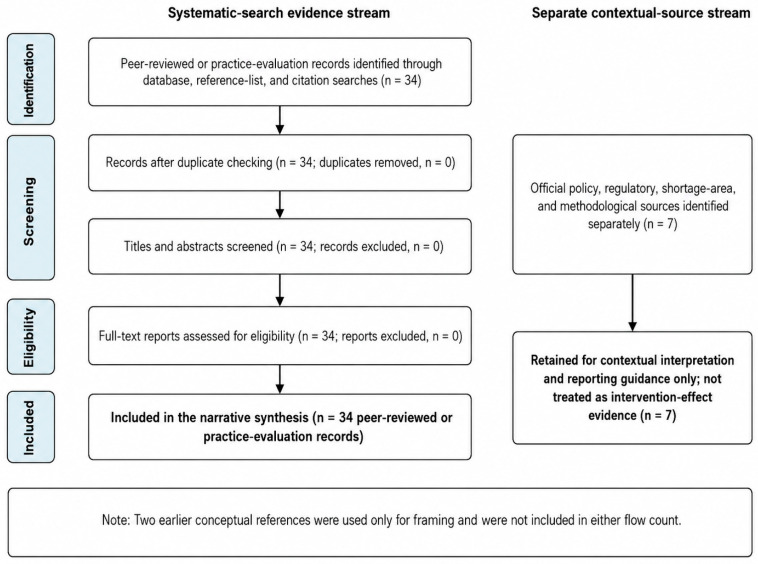
PRISMA 2020-style flow diagram separating the systematic-search evidence stream from the contextual-source stream. Thirty-four peer-reviewed or practice-evaluation records were included in the narrative synthesis; seven official sources were retained only for contextual interpretation and reporting guidance. Two earlier conceptual references used for framing were not included in either flow count [21].

D.K. extracted study design, jurisdiction, population, rural/suburban/frontier context, pharmacist role, physician or prescriber coordination mechanism, access outcomes, chronic disease or medication-management outcomes, emergency-department or health-system utilization outcomes, implementation observations, and reported limitations using a structured framework. T.K. and J.L.M.S. reviewed the extracted information during formal analysis and narrative coding. No study investigators were contacted, and no missing values were imputed. Where comparable summary statistics or effect estimates were reported, they were extracted; otherwise, findings were summarized narratively. Study selection is shown in Figure 1.

The finalized evidence set comprised 34 distinct eligible peer-reviewed or practice-evaluation records for narrative synthesis. Seven official policy, regulatory, shortage-area, or methodological sources were retained for contextual interpretation or reporting guidance but were not treated as intervention-effect studies. Two additional references were used only for conceptual framing and were not included in the flow count.

### 2.5. Quality and Certainty Assessment

Methodological appraisal used the design-appropriate Critical Appraisal Skills Programme checklist [22]. D.K. completed the initial appraisal, and T.K. reviewed the judgments during synthesis; differences were resolved by discussion. A formal agreement coefficient was not calculated. Randomized trials were assessed for allocation, follow-up, outcome measurement, and intervention fidelity; observational studies for representativeness, exposure and outcome ascertainment, confounding, and ecological limitations; qualitative studies for sampling, data collection, reflexivity, and analytical credibility; and systematic reviews for search completeness, eligibility clarity, risk-of-bias assessment, and synthesis appropriateness. Official contextual sources were checked for provenance, recency, and relevance but were not assigned intervention-effect risk-of-bias ratings. Outcome-level certainty judgments were discussed by D.K., T.K., and J.L.M.S. using GRADE domains, with attention to risk of bias, inconsistency, indirectness, and imprecision [23].

### 2.6. Synthesis

Meta-analysis was not feasible because the access indicators, intervention models, jurisdictions, study designs, and outcome definitions were heterogeneous. Narrative synthesis was organized across five evidence themes: (1) rural and suburban access barriers in Alberta; (2) the Wyoming and U.S. frontier context; (3) community pharmacies as a geographic and temporal access point; (4) pharmacist–family physician coordination in chronic disease management; and (5) common ailments, emergency-department use, and medication safety. Heterogeneity was explored narratively by jurisdiction, rurality, intervention scope, coordination mechanism, outcome type, and study design. Pooled effect estimates, sensitivity analyses, and statistical tests for reporting bias were not performed. Empirical findings were reported in the Results, while author-developed transferability and implementation considerations were reserved for the Discussion.

## 3. Results

### 3.1. Study Selection

Database, reference-list, and citation searching identified 34 peer-reviewed or practice-evaluation records. No duplicate records were identified. All 34 records were screened at title/abstract and full-text stages; none was excluded after application of the eligibility criteria, and all 34 were included in the narrative synthesis. Seven official policy, regulatory, shortage-area, or methodological sources were identified and retained in a separate contextual stream. Two earlier conceptual references used only for framing were not included in either flow count. Figure 1 summarizes these separate source streams.

### 3.2. Characteristics of the Evidence Base

The evidence base contained four overlapping bodies of literature. The first addressed primary-care access and rural–urban disparities in Canada and Alberta, including conceptual frameworks, Canadian survey data, continuity research, policy analyses, and spatial-access studies [1,2,3,4,5,6,7,8,20,24]. The second described Wyoming and U.S. frontier access through shortage-area data, rural health-policy context, and qualitative patient-experience research [9,10,11,12]. The third examined community pharmacists as access points through pharmacy accessibility, prescribing, pharmacy care clinics, common-ailment services, and chronic disease management [13,14,15,16,17,18,19,25,26,27,28,29,30,31,32,33,34,35,36]. The fourth addressed physician–pharmacist collaboration, integrated primary care, continuity, and medication safety [30,31,32,33,34,35,36,37,38,39,40,41,42]. Table 2 summarizes the evidence and its principal appraisal and transferability limitations.

No included study directly compared rural Alberta, suburban Alberta, and rural Wyoming under the same coordinated pharmacist–family physician intervention. Direct clinical evidence was strongest for blood pressure, cardiovascular risk, and chronic disease medication management. Evidence was less direct for attachment, travel burden, continuity, emergency-department substitution, and rural-versus-suburban comparative effectiveness. Wyoming findings are therefore reported as contextual evidence; transferability is considered separately in the Discussion rather than presented as an observed result.

### 3.3. Rural and Suburban Access Barriers in Alberta

Geographic and service-distribution evidence provide the strongest support for the rural Alberta access problem. Alberta spatial-access studies have shown that rural and remote communities are more likely to experience longer travel times and higher non-local utilization, particularly when seeking services outside of routine primary care [5,6]. These barriers are not simply a matter of inconvenience. For the management of chronic disease, medication titration, preventive follow-up and post-discharge care, travel burden can result in delayed assessment, less frequent monitoring, and greater use of urgent or episodic alternatives.

Suburban communities may live close to clinics or pharmacies yet remain unattached to a longitudinal clinician, unable to obtain same-day advice, or reliant on walk-in clinics, urgent care, and emergency departments. Canadian survey and Alberta policy evidence identified gaps in attachment and timely appointments despite geographic proximity [7,8], and Alberta continuity research linked greater clinic and family physician continuity with patient outcomes and utilization [20]. However, the included evidence did not isolate a standardized suburban Alberta cohort or test a coordinated pharmacy intervention specifically in suburban communities. Evidence for this setting was therefore indirect.

The rural and suburban literature used different access measures. Rural studies emphasized geography, travel, and local versus non-local use, whereas suburban access was inferred mainly from attachment, timeliness, and fragmentation. This heterogeneity limited direct rural–suburban comparison and precluded pooled analysis.

### 3.4. Wyoming and U.S. Frontier Context

Wyoming contextual data documented primary-care workforce shortages and a substantial population living in designated shortage areas [9]. The Wyoming Office of Rural Health described workforce analysis, technical assistance, collaboration, and telecommunications-enabled access functions [10]. These official sources describe system context rather than outcomes of pharmacist–family physician interventions.

Wyoming qualitative research identified trust, respectful communication, adequate time, explanations, and medication-literacy support as important patient priorities [11]. Policy commentary also identified cost and access pressures in a largely rural state [12]. These findings characterize relational and practical access barriers but do not estimate the effect of coordinated pharmacist care.

No eligible study tested the same coordinated intervention in Alberta and Wyoming, and no study estimated comparative effectiveness across the two jurisdictions. Similarities in distance, workforce pressure, and dispersed service delivery therefore represent contextual analogies only. Differences in insurance, reimbursement, prescribing authority, documentation systems, and patient cost-sharing create substantial indirectness.

### 3.5. Community Pharmacy as a Geographic and Temporal Access Point

Community pharmacies are often described as accessible because they are widely distributed, routinely used, and commonly available without appointment. United States Medicare data showed that community pharmacy visits outnumbered primary-care physician encounters among older adults and the difference was larger in rural nonmetropolitan areas than in metropolitan areas [13]. A nationwide United States geographic information systems analysis found that almost 90% of Americans lived within five miles of a community pharmacy, but rural access relied heavily on independent and regional pharmacies [14]. A later drive-time study found that rural census tracts had lower pharmacy access than suburban and urban tracts at both 10 and 20 min drive times [15].

Canadian scope literature and Alberta standards document broad medication-management and prescribing functions for pharmacists, although scope and reimbursement vary by jurisdiction [16,17,18]. Commentary and Alberta practice evaluations describe pharmacy care clinics as an emerging access model [19,26]. The available evidence, however, does not establish that the presence of a pharmacy or clinic by itself improves continuity, diagnostic safety, or health-system outcomes.

A systematic review of pharmacist prescribing reported improved access to medicines, but the included models, populations, and outcome definitions were heterogeneous [27]. Alberta pharmacy care clinic evidence described the services sought by patients, including common ailments, chronic disease management, point-of-care testing, and public health services [26]. Direct comparative evidence on attachment, continuity, travel reduction, and equity remained limited.

### 3.6. Pharmacist–Family Physician Coordination and Chronic Disease Management

The strongest intervention evidence concerned hypertension and cardiovascular risk management. In RxACTION, the intervention group had a mean systolic blood pressure reduction of 18.3 mmHg at six months compared with 11.8 mmHg in the control group; an adjusted between-group difference of 6.6 mmHg; the adjusted odds of reaching the blood pressure target were 2.32 in favour of the intervention [28]. RxEACH enrolled 723 patients and reported a 21%-greater reduction in estimated cardiovascular risk with community pharmacist case finding, prescribing, and follow-up [29]. In a U.S. physician–pharmacist trial, 62% of intervention participants reached target blood pressure compared with 44% receiving usual care [30]. SCRIP-HTN reported an adjusted 5.6 mmHg greater systolic blood pressure reduction at six months with a community pharmacist–nurse intervention [31].

Systematic reviews and meta-analyses also reported improvements in blood pressure, glycemic control, and other chronic disease outcomes from pharmacist-led or pharmacist-collaborative interventions [32,33,34,37]. The contributing interventions varied in pharmacist authority, physician involvement, follow-up intensity, population, and setting. This evidence supports medication and chronic disease functions more strongly than it supports any single Alberta–Wyoming service model.

U.S. integrated pharmacist–primary-care models reported favourable clinical or utilization outcomes in physician-linked settings [38,39]. Because these models were often embedded in clinics or federally qualified health centres rather than independent community pharmacies, their relevance to rural community pharmacy pathways was considered indirect.

### 3.7. Common Ailments, Emergency-Department Use and Medication Safety

Systematic review evidence indicates that pharmacy-based minor-ailment schemes can substitute for some encounters with other providers, although definitions, scope, and outcome measures vary across programmes [35]. Medicine-optimization evidence also supports pharmacist-led identification of medication-related problems, but the included initiatives were heterogeneous [36]. Neither body of evidence established that substitution necessarily improves continuity or reduces emergency-department use.

An Alberta community pharmacy care clinic evaluation described substantial use for common ailments, chronic disease management, point-of-care testing, and public health services [26]. A separate Alberta-informed publication proposed an ICD-10 framework for classifying potentially pharmacy-sensitive emergency-department visits [25]. The latter was an evaluation framework rather than a direct test of emergency-department reduction; certainty for emergency-department impact therefore remained low to very low.

Canadian studies documented medication-related emergency-department visits, drug-related hospitalizations, and clinically important drug–drug interactions [40,41,42]. These studies establish medication harm as an important outcome, but they do not directly quantify the effect of expanded community pharmacist prescribing or coordinated pharmacist–family physician care on such harms.

### 3.8. Methodological Appraisal and Certainty of Evidence

Across randomized trials, strengths included prospective designs and objective clinical outcomes; common limitations included limited blinding, intervention heterogeneity, and setting-specific delivery. Observational access studies were limited by cross-sectional or retrospective designs, residual confounding, and ecological measures. Qualitative Wyoming evidence was useful for patient experience but did not estimate intervention effects. Systematic reviews varied in included models and outcomes, contributing inconsistency and indirectness. Table 2 summarizes these appraisal considerations. Outcome-level certainty was moderate to high for pharmacist effects on hypertension and cardiovascular risk management; moderate for community pharmacists as frequent patient-contact points; low to moderate for medicine access and common-ailment substitution; low to very low for continuity and emergency-department effects; and very low for equivalent effectiveness across rural Alberta, suburban Alberta, and rural Wyoming. Table 3 summarizes the certainty judgments and their main limitations.

## 4. Discussion

### 4.1. Principal Interpretation

Taken together, the evidence supports coordinated pharmacist involvement for selected medication-related and chronic disease outcomes, particularly blood pressure and cardiovascular risk. Community pharmacies also provide frequent and often geographically accessible contact points. Evidence is weaker for attachment, continuity, emergency-department use, and equity, and the review does not establish that one model is equally effective in rural Alberta, suburban Alberta, and rural Wyoming.

The practical implication is that pharmacists may extend, rather than replace, physician-led primary care when roles are clearly defined and communication is bidirectional. Primary-care clinicians retain responsibility for diagnosis, complex care, longitudinal care plans, and escalation, while pharmacists may contribute medication assessment, adaptation or renewal where authorized, chronic disease monitoring, adherence support, and early identification of red flags. This interpretation is consistent with team-based primary-care-capacity literature [43], but it still requires prospective evaluation in each setting.

### 4.2. Alberta–Wyoming Transferability: Contextual Interpretation

The Alberta–Wyoming comparison is contextual rather than a direct comparative effectiveness analysis. The settings share some practical constraints, including rural distance, dispersed communities, workforce pressure, and dependence on local professional relationships. They also differ in public versus mixed insurance arrangements, reimbursement, pharmacist prescribing authority, collaborative practice rules, documentation systems, and patient cost-sharing. An intervention that works in Alberta therefore cannot be assumed to have the same reach, uptake, safety, or effect in Wyoming.

Transferability is most plausible for mechanisms supported across several settings, such as medication review, blood pressure monitoring, cardiovascular risk management, adherence support, and timely referral. It is less certain for emergency-department diversion, attachment, and continuity because direct outcome evidence is sparse and these outcomes depend heavily on local workflows and information exchange.

Table 4 presents an author-developed framework for considering transferability. It is intended to identify components and outcomes for prospective evaluation, not to report observed equivalence between jurisdictions.

Any Wyoming implementation should therefore be locally adapted and evaluated rather than imported wholesale from Alberta. This is an interpretation of the evidence and context, not an empirical finding from the included studies.

### 4.3. Proposed Coordinated Care Model for Prospective Evaluation

A proposed model would link the patient and family, community pharmacist, and family physician or primary-care clinic through defined responsibilities and shared coordination processes. Pharmacist functions could include assessment of specified medication-related problems and common ailments, medication review, adaptation or renewal where authorized, chronic disease monitoring, adherence support, and referral. Physician or clinic functions would include diagnosis, complex care, longitudinal care-plan ownership, follow-up, and escalation. Encounters, medication changes, follow-up requirements, and red flags would be documented and communicated to the clinic of record. This is a model for prospective testing rather than an effect demonstrated by the review; its components are summarized in Table 5.

### 4.4. Implementation Considerations

Implementation should begin with the access problem a community is trying to address. Rural Alberta models may focus on reducing travel for medication review, blood pressure checks, uncomplicated common ailments, or follow-up after medication changes. Suburban Alberta models may focus on appointment timeliness, medication continuity, and reducing disconnected walk-in care. In Wyoming and other U.S. frontier settings, implementation would also need to account for collaborative practice authority, payer requirements, patient cost-sharing, and pharmacy sustainability.

Across settings, important conditions include pharmacist workflow capacity, private consultation space, reliable documentation, sustainable reimbursement, manageable communication processes for primary-care clinicians, explicit red-flag and referral thresholds, and clear follow-up responsibility. Patients should be told that pharmacy access supplements rather than replaces longitudinal primary care. These safeguards are supported by medication-safety and continuity concerns, but their effectiveness should be measured rather than assumed.

Evaluation should be stratified by geography and attachment and, where ethically and appropriately available, by age, sex, income, Indigenous identity, immigration status, disability, and payer status. Relevant outcomes include access, continuity, clinical control, medication safety, appropriate emergency referral, patient experience, equity, and cost.

### 4.5. Strengths and Limitations

Strengths of this review include a predefined question, explicit eligibility criteria, searches across five databases, full search strings, design-appropriate CASP appraisal, GRADE certainty domains, and PRISMA 2020 reporting. The review also separated 34 synthesis records from seven contextual or methodological sources and distinguished empirical findings from author-developed transferability and implementation considerations. The cross-jurisdictional author team contributed Alberta primary-care and community pharmacy perspectives and Wyoming rural/frontier health perspectives.

The main limitation is indirectness. No study directly compared the same coordinated intervention across rural Alberta, suburban Alberta, and rural Wyoming. Many intervention studies measured clinical markers rather than attachment, travel burden, continuity, or emergency-department substitution; most Alberta access studies did not isolate suburban populations; and Wyoming evidence was mainly contextual or qualitative. Although the review was registered with PROSPERO (CRD420261414639), the full internal protocol was not separately published, and a formal inter-reviewer agreement statistic was not collected. Because meta-analysis was inappropriate, the synthesis relied on narrative interpretation across heterogeneous designs. Reporting-bias tests and sensitivity analyses were not performed. These limitations require cautious interpretation and prospective validation.

### 4.6. Research Implications

Future studies should prospectively test coordinated pathways using pragmatic trials, stepped-wedge implementation studies, interrupted-time-series analyses, or linked administrative-data designs. Investigators should prespecify whether the primary objective is access, clinical control, continuity, medication safety, emergency-department use, equity, or cost-effectiveness, and should stratify analyses by rural, remote, suburban, metropolitan, and frontier residence.

Priority outcomes include primary-care attachment and continuity; same- or next-day access; travel time and local versus non-local care; emergency-department visits for potentially pharmacy-manageable primary-care-sensitive conditions; medically appropriate referrals; medication-related emergency visits or hospitalizations; chronic disease markers; patient-reported access and trust; provider communication; equity; and cost-effectiveness. Using parallel outcome definitions in Alberta and Wyoming could support cross-jurisdiction learning without implying that effects are interchangeable.

## 5. Conclusions

This review found the strongest evidence for coordinated pharmacist contributions to hypertension, cardiovascular risk reduction, medication management, and chronic disease monitoring. Community pharmacies may provide frequent and locally accessible contact points, but evidence is less certain for attachment, continuity, emergency-department impact, and equity. Rural Alberta, suburban Alberta, and rural Wyoming share some access constraints, yet no included study directly compared the same coordinated intervention across these settings. Relevance to Wyoming should therefore be treated as a context-dependent proposition requiring local adaptation, clear clinical boundaries, shared documentation, referral and red-flag protocols, sustainable workflows, and prospective evaluation. Coordinated pharmacist–family physician care is best understood as a potential extension of physician-led longitudinal primary care, not as a replacement for family physicians.

## Figures and Tables

**Table 1:** Eligibility criteria for the systematic review.

Domain	Inclusion Criteria	Exclusion Criteria
Population	Adults, families or general community populations living in rural, remote, suburban, peri-urban, small urban, frontier or metropolitan settings, with priority given to Alberta, Canadian and Wyoming/U.S. frontier evidence.	Pediatric-only studies unless the access model applied to whole-community primary care; inpatient-only cohorts; studies without community access relevance.
Intervention/exposure	Community pharmacist-led, pharmacist-integrated or pharmacist–family physician coordinated care; pharmacist prescribing; medication review; chronic disease monitoring; common-ailment care; point-of-care testing; referral; telepharmacy; collaborative practice agreements; shared care plans.	Dispensing-only studies, product-supply-only papers, pharmacy workforce papers without patient-care implications, or models not linked to primary-care access.
Comparator/context	Usual care; physician- or nurse-practitioner-centred care; local versus non-local care; rural versus suburban/urban access; pre–post implementation of pharmacy services; U.S. frontier contexts. Official contextual sources were classified separately from intervention-effect evidence.	Studies with no interpretable comparator, access context, coordination mechanism or implementation relevance.
Outcomes	Attachment, appointment timeliness, travel distance/time, local care use, emergency-department or urgent-care use, medication access, medication safety, chronic disease markers, cardiovascular risk, patient experience, continuity, equity and implementation outcomes.	Studies reporting only attitudes or awareness without access, service-use, clinical, safety or implementation outcomes.
Designs	Systematic reviews, randomized trials, cohort studies, cross-sectional studies, routine-data studies, qualitative and mixed-methods studies, and practice evaluations. Official policy, regulatory, shortage-area, and reporting sources were retained separately for contextual interpretation.	Narrative opinion pieces without evidence unless used only for context; editorials without implementation relevance; animal or laboratory studies.
Publication window	Peer-reviewed evidence and selected official sources from 1 January 2010 to 19 April 2026; earlier landmark primary-care and medication-safety studies were retained when necessary for conceptual framing.	Superseded policy documents, non-English texts that could not be reliably interpreted, or conference abstracts without sufficient data unless describing a very recent Alberta pharmacy access model.

**Table 2:** Summary of included evidence and key contextual sources, with principal appraisal and transferability limitations.

Study/Source	Design and Setting	Evidence Relevant to the Review	Principal Appraisal or Transferability Limitation
Levesque et al., 2013 [1]	Conceptual framework for patient-centred access.	Defines access through health-system dimensions and population abilities.	Conceptual framework; does not estimate intervention effects.
Kiran et al., 2024 [7]	Canadian cross-sectional survey of primary-care experiences.	Examined attachment, urgent appointment access and patient priorities.	Self-reported national data; not a pharmacist intervention or Alberta-specific suburban analysis.
Jacobs et al., 2025 [8]	Alberta policy and workforce analysis.	Estimated workforce requirements associated with unmet primary-care need.	Policy modelling; does not evaluate coordinated pharmacist care.
McDonald et al., 2024 [20]	Alberta retrospective continuity study.	Examined clinic and family physician continuity in relation to outcomes and utilization.	Observational and potentially confounded; not pharmacy-specific.
Liu et al., 2022 [5,6]	Alberta observational spatial-access studies in osteoarthritis.	Measured rural–urban patterns in local care use and realized travel times.	Disease-specific and non-interventional; indirect for coordinated pharmacist care.
HRSA and Wyoming Office of Rural Health [9,10]	Official shortage-area and rural-health contextual sources.	Documented Wyoming HPSA burden and state rural-health functions.	Contextual sources; not intervention-effect evidence and excluded from GRADE ratings.
Singh et al., 2018 [11]	Wyoming qualitative focus-group study.	Identified rural patient expectations, trust, communication and engagement barriers.	Qualitative and setting-specific; no intervention-effect estimate.
Bodenheimer and Smith, 2013 [43]	U.S. primary-care capacity policy analysis.	Proposed team-based redistribution of selected primary-care functions.	Conceptual policy analysis; does not test a specific coordinated model.
Berenbrok et al., 2020 [13]	U.S. Medicare cross-sectional study.	Compared community pharmacy visits with primary-care physician encounters.	Older Medicare population; encounter frequency does not establish quality, continuity or outcomes.
Berenbrok et al., 2022; Sharareh et al., 2024 [14,15]	U.S. national GIS and drive-time analyses.	Mapped pharmacy proximity and drive-time access by rurality.	Geographic availability does not establish clinical integration or effectiveness.
Tannenbaum and Tsuyuki; Raiche et al. [16,17]	Canadian pharmacist-scope and policy literature.	Described expanded pharmacist roles and Canadian scope variability.	Descriptive evidence; regulatory context varies substantially across jurisdictions.
Ramrattan et al.; Al Hamarneh et al. [25,26]	Alberta emergency-department evaluation framework and pharmacy care clinic practice evaluation.	Proposed an ED classification framework and described services used at a pharmacy care clinic.	One source is conceptual; the practice evaluation does not establish causal ED reduction.
Walpola et al., 2024 [27]	Systematic review of pharmacist prescribing and medicine access.	Synthesized effects of pharmacist prescribing on access to medicines.	Heterogeneous models, outcomes and jurisdictions.
Chaudhri et al., 2023 [37]	Systematic review and meta-analysis of GP–pharmacist collaboration.	Examined effects of bidirectional collaboration on cardiovascular risk factors.	Intervention content and practice settings varied; not specific to rural Alberta or Wyoming.
Tsuyuki et al.; Hunt et al.; McLean et al. [28,29,30,31]	Randomized pharmacist or team-based chronic disease trials.	RxACTION: Adjusted systolic blood pressure difference 6.6 mmHg; RxEACH: 21%-greater reduction in estimated cardiovascular risk; Hunt et al.: 62% versus 44% reached target; SCRIP-HTN: 5.6 mmHg greater systolic blood pressure reduction.	Strongest causal evidence, but blinding was limited and intervention models and settings varied.
Cheema et al.; Coutureau et al.; Newman et al. [32,33,34]	Systematic and umbrella reviews of pharmacist chronic disease interventions.	Synthesized hypertension, diabetes and broader chronic disease outcomes.	Substantial heterogeneity in interventions, populations and coordination intensity.
Paudyal et al.; Chambers et al. [35,36]	Systematic reviews of minor ailments and medicine optimization.	Evaluated substitution, medicine optimization and overprescribing initiatives.	Programme definitions and outcomes varied; continuity and ED effects remain uncertain.
Matzke et al.; Rodis et al. [38,39]	U.S. collaborative care and federally qualified health centre models.	Evaluated pharmacist integration in physician-linked primary-care settings.	Often clinic-embedded rather than independent community pharmacy; setting-specific.
Zed et al.; Samoy et al.; Juurlink et al. [40,41,42]	Canadian medication-safety studies.	Documented medication-related ED visits, hospitalizations and drug–drug interaction risks.	Mostly older observational evidence; establishes burden rather than the effect of expanded pharmacist roles.

**Table 3:** CASP-informed GRADE summary of outcome-level certainty and principal limitations.

Finding	Certainty	Basis and Principal Limitations
Rural Alberta residents experience greater geographic barriers to local primary care and related services than metropolitan residents.	Moderate	Supported by Alberta spatial-access studies and Canadian rural health literature; magnitude varies by service, community and condition.
Suburban residents can face important access barriers even when geographic proximity is better.	Low to moderate	Supported by Canadian survey, continuity and Alberta policy evidence; Alberta-specific suburban stratification remains limited.
Wyoming shares some rural access constraints with Alberta.	Low	Supported by official shortage-area data and qualitative Wyoming evidence; no direct intervention comparison and major system differences create indirectness.
Community pharmacists are frequent and often geographically accessible patient-contact points.	Moderate	Supported by U.S. Medicare and geographic-access studies; rural pharmacy availability remains vulnerable and access does not establish integration.
Pharmacist–family physician collaboration improves chronic disease and cardiovascular risk outcomes.	Moderate to high	Supported by randomized trials and systematic reviews, especially for blood pressure and cardiovascular risk factors.
Pharmacy-based common-ailment pathways can substitute for some encounters with other providers.	Low to moderate	Supported by systematic review evidence, but programmes, scope, triage and communication processes vary.
Community pharmacy care clinics reduce emergency-department use.	Very low	Supported mainly by an evaluation framework and service-use evidence; direct causal ED outcome evidence is unavailable.
Coordinated pharmacist access improves continuity when linked with shared documentation and referral pathways.	Low	Plausible and indirectly supported by continuity and coordination evidence; few direct comparative studies are available.
Comparable effectiveness across rural Alberta, suburban Alberta and rural Wyoming.	Very low	No direct comparative study; substantial indirectness related to geography, staffing, reimbursement, scope and referral infrastructure.

**Table 4:** Author-developed transferability considerations for Alberta and Wyoming/frontier U.S. settings.

Domain	Alberta Evidence or Context	Wyoming/U.S. Frontier Context	Question for Prospective Evaluation
Primary access problem	Rural: Distance, local service fragility and workforce gaps. Suburban: Attachment, appointment timeliness and fragmented episodic care.	Frontier/rural: HPSA designations, travel distance, small-community service dependence and workforce recruitment challenges.	Would a coordinated local pathway improve selected access outcomes when effects are estimated separately by geography and attachment?
Role of family physician	Longitudinal diagnosis, complex care, care-plan ownership, referral and continuity.	Primary-care physician availability may be limited in shortage areas; continuity may depend on regional or networked care.	Can physician or primary-care responsibility for diagnosis, complex care, longitudinal planning and escalation be maintained reliably?
Role of community pharmacist	Medication review, adaptation or renewal, prescribing where authorized, chronic disease monitoring, common ailments and referral.	Medication management and collaborative practice may provide local support where physician availability is limited.	Which medication-related and chronic disease functions are feasible within local scope, payment and supervision rules?
Rural emphasis	Potential reduction in travel through local monitoring, telehealth linkage and regional referral.	Frontier travel barriers and local pharmacy vulnerability make sustainability central.	What are the effects on travel burden, local care use, staffing sustainability and cross-site communication?
Suburban emphasis	Potential gains in timeliness, medication continuity and reduced fragmented episodic care.	Comparable U.S. settings may also experience insurance and network fragmentation.	Does the model improve continuity and reduce fragmentation rather than merely increasing visit volume?
Safety requirements	Red-flag screening, medication reconciliation, laboratory access where appropriate, documentation and follow-up.	Collaborative practice agreements and referral thresholds may be required where pharmacists act under protocol.	Are red-flag, medication-reconciliation, documentation, follow-up and referral processes reliable in routine practice?
Evaluation outcomes	Attachment, timeliness, travel time, local care use, ED use, medication-related ED visits, clinical markers, patient-reported access and equity.	HPSA metrics, ED or urgent-care use, pharmacy access, chronic disease control, medication safety and patient trust.	Can comparable outcome definitions be used while estimating jurisdiction-specific effects?

**Table 5:** Proposed coordinated pharmacist–family physician access model for prospective evaluation in Alberta and Wyoming/frontier U.S. settings.

Component	Proposed Coordinated Model	Rural Alberta Emphasis	Suburban Alberta Emphasis	Wyoming/Frontier U.S. Emphasis
Access point	Community pharmacy assessment for prespecified medication issues, common ailments and chronic disease monitoring.	May reduce travel and non-local care for selected services.	May improve timely assessment and reduce disconnected episodic care.	Potential first-contact option where physician access is scarce or distant.
Clinical scope	Medication review, prescribing or adaptation where authorized, point-of-care testing, immunization, chronic disease follow-up and referral.	Potentially useful where physician or nurse-practitioner access is intermittent or distant.	Potentially useful where attachment exists but timely appointments are limited.	Would require adaptation to collaborative practice, payment and supervision rules.
Continuity mechanism	Document the service, communicate medication changes, refer to the regular clinician and use shared care plans.	Could reduce isolated episodic care across small or regional services.	Could prevent pharmacy clinics from becoming another disconnected walk-in pathway.	Could support team-based care across dispersed sites if information exchange is reliable.
Safety mechanism	Red-flag screening, medication reconciliation, interaction review, follow-up plans and emergency referral criteria.	Particularly important when diagnostic resources are limited.	Particularly important in high-volume and after-hours contexts.	Critical where insurance, scope-of-practice and referral arrangements vary.
Evaluation outcomes	Attachment, timeliness, travel time, local care use, ED visits, medication-related ED visits, clinical markers, patient-reported access and equity.	Emphasize travel burden, local access and service sustainability.	Emphasize timeliness, fragmentation and continuity.	Emphasize HPSA-related access, pharmacy sustainability, chronic disease outcomes and ED or urgent-care use.

## Data Availability

No new data were created or analyzed in this study. The synthesis is based on the published literature and official sources cited in the manuscript; the extraction fields are described in Section 2.4.

## Supplementary Materials

The following supporting information can be downloaded at https://www.mdpi.com/article/10.3390/pharmacy14040098/s1, Supplementary File S1: PRISMA 2020 checklist [21].

## Abbreviations

CASP: Critical Appraisal Skills Programme; ED: Emergency Department; GRADE: Grading of Recommendations Assessment, Development and Evaluation; HPSA: Health-Professional Shortage Area; PRISMA: Preferred Reporting Items for Systematic Reviews and Meta-Analyses; U.S.: United States.
